# Global research priorities for social, behavioural and community engagement interventions for maternal, newborn and child health

**DOI:** 10.1186/s12961-020-00597-7

**Published:** 2020-08-27

**Authors:** Geoffrey Chan, J. Douglas Storey, Manoja Kumar Das, Emma Sacks, Mira Johri, Tamar Kabakian-Khasholian, Deepak Paudel, Sachiyo Yoshida, Anayda Portela

**Affiliations:** 1 Burnet Institute, Melbourne, Australia; 2 Johns Hopkins Bloomberg School of Public Health, Center for Communication Programs, Baltimore, United States of America; 3 The INCLEN Trust International, New Delhi, India; 4 Department of Research, Elizabeth Glaser Pediatric AIDS Foundation, Washington, DC United States of America; 5 Centre de Recherche du Centre Hospitalier de l’Université de Montréal (CRCHUM), Montréal, Canada; 6 Department of Health Promotion and Community Health, Faculty of Health Sciences, American University of Beirut, Beirut, Lebanon; 7 Save the Children, Kathmandu, Nepal; 8 Department of Maternal, Newborn, Child and Adolescent Health and Ageing, World Health Organization, Avenue Appia 20, 1202 Geneva, Switzerland

**Keywords:** Research priority, maternal health, newborn health, child health, health promotion, social and behavioural change, community engagement, CHNRI

## Abstract

**Background:**

Social, behavioural and community engagement (SBCE) interventions are essential for global maternal, newborn and child health (MNCH) strategies. Past efforts to synthesise research on SBCE interventions identified a need for clear priorities to guide future research. WHO led an exercise to identify global research priorities for SBCE interventions to improve MNCH.

**Methods:**

We adapted the Child Health and Nutrition Research Initiative method and combined quantitative and qualitative methods to determine MNCH SBCE intervention research priorities applicable across different contexts. Using online surveys and meetings, researchers and programme experts proposed up to three research priorities and scored the compiled priorities against four criteria – health and social impact, equity, feasibility, and overall importance. Priorities were then ranked by score. A group of 29 experts finalised the top 10 research priorities for each of maternal, newborn or child health and a cross-cutting area.

**Results:**

A total of 310 experts proposed 867 research priorities, which were consolidated into 444 priorities and scored by 280 experts. Top maternal and newborn health priorities focused on research to improve the delivery of SBCE interventions that strengthen self-care/family care practices and care-seeking behaviour. Child health priorities focused on the delivery of SBCE interventions, emphasising determinants of service utilisation and breastfeeding and nutrition practices. Cross-cutting MNCH priorities highlighted the need for better integration of SBCE into facility-based and community-based health services.

**Conclusions:**

Achieving global targets for MNCH requires increased investment in SBCE interventions that build capacities of individuals, families and communities as agents of their own health. Findings from this exercise provide guidance to prioritise investments and ensure that they are best directed to achieve global objectives. Stakeholders are encouraged to use these priorities to guide future research investments and to adapt them for country programmes by engaging with national level stakeholders.

## Background

The United Nations Sustainable Development Goals 2030 [1], the Every Woman Every Child Global Strategy for Women’s, Children’s, Adolescents' Health (2016–2030) (EWEC Global Strategy) [2] and the Declaration of Astana on Primary Health Care [3] underscore the need to transform societies and governance in ways that support “*women, children and adolescents*” as “*potentially the most powerful agents for improving their own health and achieving prosperous and sustainable societies*” [2]. The implementation of effective social, behavioural and community engagement (SBCE) interventions is critical to achieving this ambition.

**Fig. 1:**
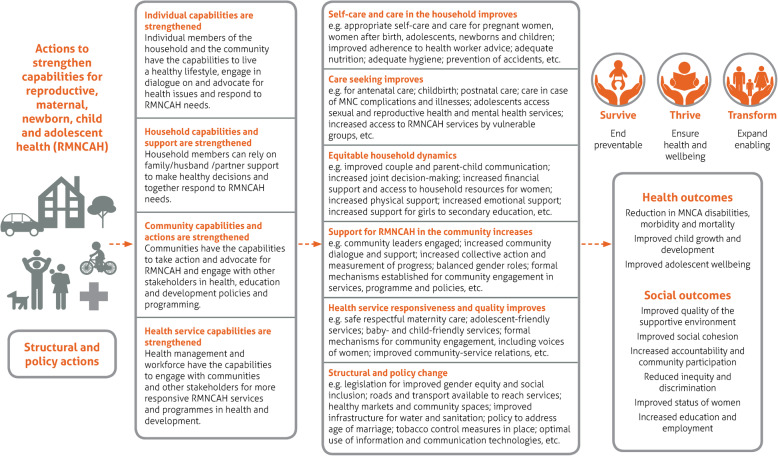
Conceptual framework for SBCE interventions for women, children’s and adolescents’ health [2]

The term ‘SBCE interventions’ encompasses a range of interventions that aim to strengthen the capabilities of individuals, families and communities to contribute to improving their own health [4]. SBCE interventions are also considered critical for addressing the social, economic, cultural and structural conditions that create an enabling environment for good health and the EWEC Global Strategy objectives of Survive, Thrive and Transform [4]. The conceptual framework in Fig. 1 presents how these interventions contribute to the health of women, children and adolescents in the EWEC Global Strategy [2]. Other terms that are commonly used for what we refer to as SBCE interventions include health promotion, demand creation, empowerment, and social and behaviour change [4].

While SBCE interventions are essential to achieving good maternal, newborn and child health (MNCH), they are undervalued in current global MNCH policy and programming [4–6]. Underlying this are fundamental gaps in knowledge about which SBCE interventions will have the desired impact and how they can best be implemented [4–6]. Concerted effort is required to ensure research addresses these gaps. Global research priorities have recently been set for maternal and perinatal health [7], newborn health [8, 9], and adolescent health [10]; some SBCE research priorities were identified within these exercises. However, a research agenda specifically for SBCE interventions for MNCH could better direct research towards the most important gaps in this field. Hence, the aim of this study was to define global priorities for research on SBCE interventions to improve MNCH. This paper presents the priorities that were identified through a consultative exercise managed by WHO.

## Methods

### Study design

Concurrent priority setting exercises were conducted for three health areas – (1) maternal, (2) newborn and (3) child health. Adolescent health was not included because a priority setting exercise had recently been conducted and the results included questions that pertained to SBCE interventions [10]. A mixed-methods approach was used, which combined an adapted version of the Child Health and Nutrition Research Initiative (CHNRI) methodology [11] with a qualitative, consensus-building approach with experts. The CHNRI method features a metrics-based approach in which subject experts score a set of research priorities to determine their relative importance and has been used in over 50 health research prioritisation exercises [7–12].

**Fig. 2:**
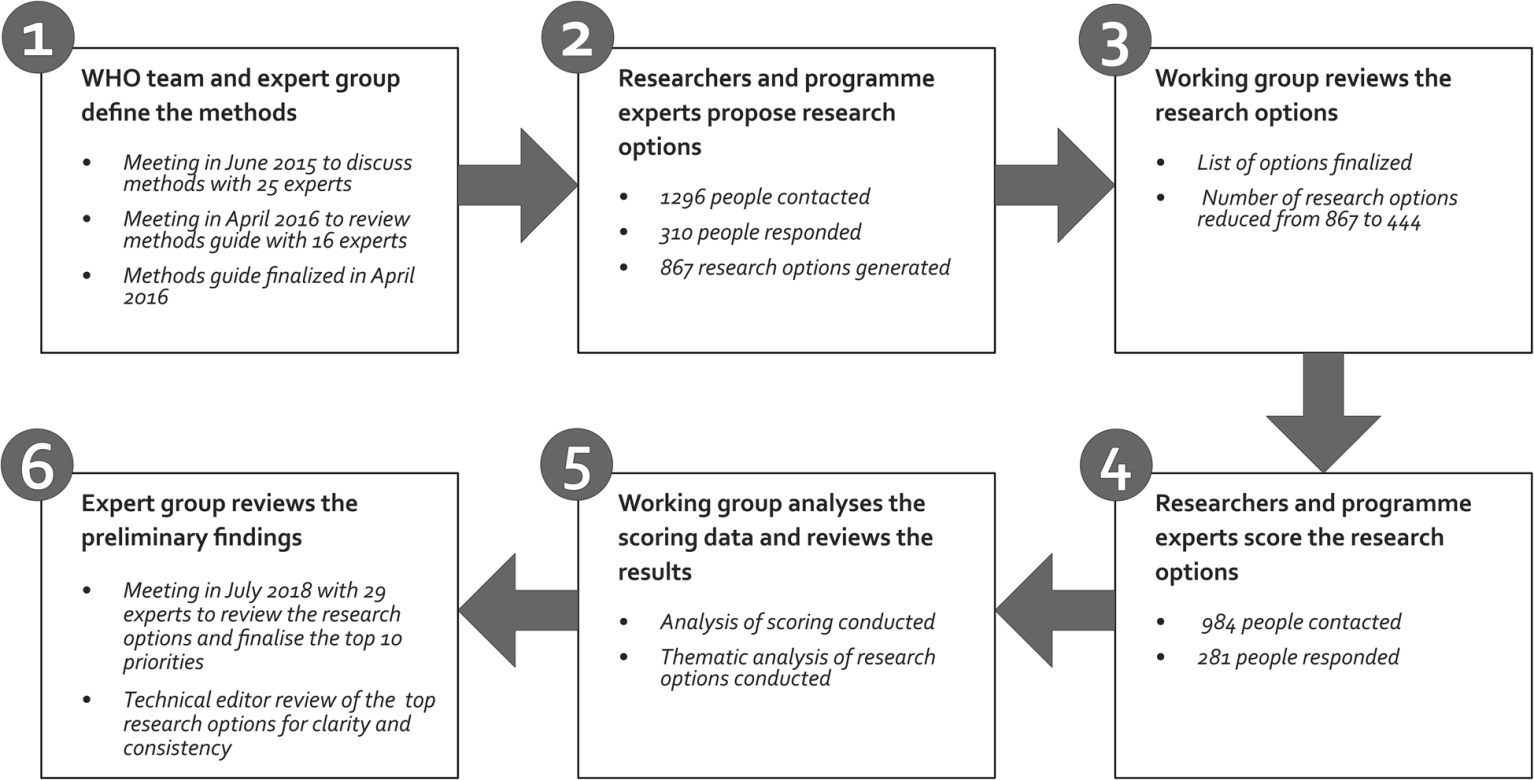
The six steps in the research priority setting exercise for SBCE interventions for MNCH

The exercise was conducted between June 2015 and August 2018. Figure 2 outlines the six main steps followed. In step 1, beginning in June 2015, meetings of partners were held to discuss the methods. The methods were finalised in 2016 in a consultation meeting organised by WHO with 25 SBCE researchers and practitioners. A methods guide was developed and peer reviewed (available from the corresponding author upon request). Data collection occurred between August 2016 and December 2017: technical experts proposed SBCE research priorities (step 2); the WHO working group collated and cleaned the research priorities (step 3); and technical experts scored SBCE research priorities according to criteria (step 4). Data analysis (steps 5 and 6) was completed in July 2018 when 29 technical experts met to review the preliminary findings and to agree on the top 10 SBCE research priorities for each health area. Details of each step are explained further below.

### Participants in the proposal and scoring surveys

In step 2 (Fig. 2), participants representing two categories — researchers and programme experts — were purposively selected for each of the three health areas. Programme experts were further sub-categorised as shown in Box 1.

Researchers and programme experts were identified through database searches (PubMed and Web of Science), reference lists of relevant publications, web searches, professional networks and organisation member lists. Experts were invited to participate via email. The composition of the group of experts invited to participate incorporated a balance between high- and low- to middle-income countries, WHO regions, and male and female participants. Experts were also encouraged to forward the invitation to other suitable experts. Additional experts were identified and invited to replace those who could not be contacted due to invalid email addresses. In step 4 (Fig. 2), all participants previously contacted in step 2 were invited to score the research priorities for their health area. The expert group for the final meeting (step 6) was made up of experts from across the different categories. They were purposively selected by WHO based on expert knowledge of SBCE research and/or programmes. Efforts were made to ensure gender balance and geographic representation. They were not required to have participated in the previous steps.

Experts were contacted in their professional capacity and participation was voluntary. Basic demographic information was requested (sex, country of work and type of organisation) and all data were de-identified in the analysis to protect confidentiality.

### Data collection

#### Proposal of SBCE research priorities

In step 2, experts were invited to propose up to three research priorities for one health area (either maternal or newborn or child health) that would be relevant for the current period to 2030. Experts received an information brief that outlined the purpose and scope of the exercise, the methodology, a framework for proposing research priorities (Table 1), and instructions for proposing research priorities. The purpose of the framework was twofold: to make apparent that research pertaining to any of the three objectives of the EWEC Global Strategy (objectives of Survive, Thrive and Transform) was of interest and to encourage experts to think about the specific type of research that was needed from among the four CHNRI research domains (description, development, delivery and discovery). Experts were asked to review the framework and to try to formulate their research priorities to specify with which of the EWEC Global Strategy objectives and which of the four CHNRI research domains the priority aligned.

**Table 1:** Framework for proposing SBCE intervention research priorities

CHNRI research domains [11]	EWEC Global Strategy objectives [2]		
**What kind of research is needed?**	**Survive**	**Thrive**	**Transform**
	Interventions that save lives	Interventions that improve health status	Interventions that sustain health and social outcomes
**Discovery** **Find new approaches**	Find a new approach to save lives	Find a new approach to improve health status	Find a new approach to sustain health and social outcomes
**Development** **Make interventions simpler or cheaper**	Make a life-saving intervention simpler or cheaper	Make a health improvement intervention simpler or cheaper	Make a sustainability intervention simpler or cheaper
**Delivery** **Scale-up or increase impact of existing approaches**	Scale-up or improve the impact of a life-saving intervention	Scale-up or improve the impact of a health improvement intervention	Scale-up or improve the impact of a sustainability intervention
**Descriptive** **Understand the health challenge better**	Understand factors that improve survival	Understand factors that improve health status	Understand factors that sustain social and health outcomes

*CHNRI* Child Health and Nutrition Research Initiative, *EWEC* Every Woman Every Child, *SBCE* social, behavioural and community engagement

Participants were asked to propose research options that could be applicable for different contexts and that: (1) targeted a timeframe of 3–5 years as the period in which the research could be conducted; (2) addressed interventions, outcomes, populations, contexts and health issues relevant to the respective health area; and (3) were sufficiently broad to enable a range of specific research questions to be generated within each priority. Table 2 shows how research options compare to other levels of detail for defining research priorities [11].

**Table 2:** Levels of detail for defining research priorities

Level of detail	Examples
Health area	Maternal health
Research avenue	Research to identify social, behavioural and community engagement interventions that contribute to improved maternal health and rights
Research option	Research on interventions to make health services more accountable and responsive to community needs for improved maternal health
Research question	Does community monitoring of health services influence health facility performance for maternal health?
	Is there a set of indicators on health facility performance that will lead facilities to improve their services if the data is routinely made available to the public?

The invitation and accompanying materials were made available in Spanish and French to participants requesting translated versions.

In step 3, the submitted research priorities were compiled and organised thematically by the lead authors (GC and AP). Submissions were merged if they overlapped or there were duplicate priorities. They were also reworded for consistency and clarity and redrafted so that the priorities all had a similar scope and level of detail. Priorities were excluded if they were not relevant to SBCE interventions (e.g. clinical interventions), not related to MNCH, or if they only indicated a CHNRI research domain or an EWEC objective but had no link to SBCE interventions or outcomes. Experts who proposed relevant priorities but did not specify the CHNRI research domain were contacted for clarification. The development and delivery research domains were collapsed into a single domain since many submissions spanned both. This reduced the research domains to three. Co-authors (JDS, MKD and ES) reviewed the collated set of research priorities against the original submissions to assess the validity of the actions taken by the lead authors. Any concerns were discussed and resolved. The list of research priorities for each health area was finalised after this review. Some research priorities were relevant across MNCH areas and a set of these cross-cutting priorities was included in the final lists for all three health areas.

#### Scoring of SBCE research priorities

For step 4, Lime Survey version 3.1.1 was used to prepare a web-based survey for each of the three health areas using the final lists of research priorities. Each expert was asked to score a subset of up to 30 research priorities. The subsets included priorities from all three research domains (discovery, development/delivery and descriptive). Respondents were asked to score each priority against four pre-defined scoring criteria (Box 2).

### Data analysis

Analysis of the data collected in step 5 was conducted in Stata version 13. For each research priority, mean scores were calculated for each of the four criteria (sum of scores divided by the number of responses). The sum of the mean scores for each criterion was calculated to produce a final score for each research priority. Research priorities were then ranked by the final score. A fourth cross-cutting health area was created by calculating final scores for the set of cross-cutting priorities using all scores — whether scored by maternal, newborn or child health experts. The cross-cutting research priorities were also retained in the scoring and analysis for the original three health areas.

For step 6, in July 2018, an expert group met to review the results of scoring of the priorities. Participants were assigned to sub-groups according to each of the four health areas. The sub-groups were tasked with reviewing the top 30 ranked priorities to produce a top 10 list of research priorities. It was decided that a top 10 across each of MNCH as well as the cross-cutting area would provide an ample but focussed research agenda. Moreover, the expert group felt that the top 30 ranked priorities still contained some overlap. Hence, the sub-groups were asked to merge priorities that overlapped and to revise the wording of the research priorities for clarity, consistency, specificity or scope, ensuring that the research priorities in the final top 10 were distinct from one another while still preserving the content of the priorities as they were scored. The sub-groups chose to work with themes they identified in the research priorities rather than organising them by CHNRI research domains and the EWEC objectives. Consequently, some of the final priorities spanned more than one domain or EWEC objective. The resulting top 10 priorities for each health area were reviewed by the other sub-groups to assess the validity of the actions taken and the clarity in wording. Priorities were further revised in response to queries.

The lists of the top 10 priorities proposed in the meeting were reviewed by the lead authors (AP and GC) and sent to a technical editor to ensure consistent and concise wording of each priority and that each priority represented a distinct topic.

## Results

### Participants in the proposal and scoring surveys

To elicit research priorities (step 2), 1296 people were invited to participate, of whom 23.9% (*n* = 310) proposed research priorities. To score the research priorities (step 4), invitations were sent to 940 experts and 29.9% (*n* = 281) scored the research priorities.

Table 3 shows the composition of participants by country income, gender and expert type for each of the health areas. The analysis did not determine how many participants in step 2 also participated in step 4. The cross-cutting health area was created based on the results from the scoring stage; therefore, characteristics were only determined for that stage.

**Table 3:** Characteristics of experts who participated in proposing and scoring priorities

Health area	Characteristics		Experts who proposed research priorities		Experts who scored research priorities	
			No.	%	No.	%
**Maternal**	Total		120		78	
	Sex	Female	76	63.3	48	61.5
		Male	39	32.5	30	38.5
		Not recorded	5	4.2	0	0.0
	Type of expert	Programme	83	69.2	53	67.9
		Researcher	37	30.8	25	32.1
	Income level of country of residence^a^	HIC	50	41.7	35	44.9
		LMIC	70	58.3	43	55.1
**Newborn**	Total		109		116	
	Sex	Female	54	49.5	63	54.3
		Male	54	49.5	53	45.7
		Not recorded	1	0.9	0	0.0
	Type of expert	Programme	80	73.4	70	60.3
		Researcher	29	26.6	45	38.8
		Not recorded	0	0.0%	1	0.9
	Income level of country of residence^a^	HIC	55	50.5	51	44.0
		LMIC	52	47.7	63	54.3
		Not recorded	2	1.8	2	1.7
**Child**	Total		81		87	
	Sex	Female	43	53.1	52	59.8
		Male	36	44.4	32	36.8
		Other	0	0.0	1	1.1
		Not recorded	2	2.5	2	2.3
	Type of expert	Programme	51	63.0	56	64.4
		Researcher	29	35.8	29	33.3
		Not recorded	1	1.2	2	2.3
	Income level of country of residence^a^	HIC	36	44.4	40	46.0
		LMIC	42	51.9	45	51.7
		Not recorded	3	3.7	2	2.3
**Cross-cutting**	Total				240	
	Sex	Female			139	57.9
		Male			100	41.7
		Other			1	0.4
	Type of expert	Programme			154	64.2
		Researcher			86	35.8
	Income level of country of residence^a^	HIC			106	44.2
		LMIC			133	55.4
		Not recorded			1	0.4

^a^Based on the 2018 World Bank Classification. *HIC* high-income country, *LMIC* low- and middle-income country

### Research priorities proposed and scored

A total of 867 research priorities were proposed: 333 for maternal health, 303 for newborn health and 231 for child health. After review and consolidation, a total of 444 research priorities were sent to experts for scoring. Table 4 shows the number proposed in step 2 and the number scored in step 4 by health area.

**Table 4:** Number of research priorities proposed and scored, by health area

	Maternal	Newborn	Child	Total
Number of priorities proposed by experts in step 2	333	303	231	867
Number of priorities sent to experts to score in step 4^a^	146	163	135	444

^a^Includes 57 cross-cutting priorities

Table 5 summarises the number of times research priorities were scored by health area once subsets of priorities were randomly allocated to scorers.

**Table 5:** Number of times research priorities were scored

		Number of times individual research priorities were scored		
Health area	Number of scorers	Maximum	Minimum	Mode
Maternal	78	17	7	12
Newborn	116	25	10	16
Child	87	21	12	14
Cross-cutting	240	55	15	43

### Top 10 SBCE research priorities per health area

The top 10 research priorities by health area are presented in Tables 6, 7, 8 and 9. The lists of top 30 priorities by research domain and EWEC objective are available from the corresponding author.

**Table 6:** Top 10 maternal health SBCE research priorities

Rank	Maternal health research priority
1	Understand the determinants of self-care, family care practices, utilisation of maternity care services and access to life-saving commodities, and improve the delivery of approaches to address these determinants
2	Find new approaches and improve the delivery of existing approaches to increase community awareness, capacity, mobilisation, engagement and social accountability
3	Improve the delivery of approaches to strengthen health service responsiveness to cultural needs and preferences and to strengthen intercultural linkages between the health services and the community
4	Find new approaches and improve the delivery of existing approaches to foster effective male involvement in efforts to increase maternal, newborn and child well-being and access to care
5	Improve the delivery of health education and counselling interventions that are tailored to the needs of different audiences to improve self-care, family care practices and utilisation of maternity care services
6	Improve the delivery of community-based midwifery programmes to foster individual, family and community behaviours that improve maternal and newborn health
7	Find new approaches and improve the delivery of existing approaches to increase access to and use of modern family planning methods, including emergency and post-abortion contraception for women and adolescent girls
8	Understand the determinants of gender inequity and find new approaches to foster women’s empowerment and agency to improve their health
9	Find new approaches to address hunger and malnutrition
10	Improve the delivery of approaches to increase the uptake of HIV prevention and care services during pregnancy, birth and the postnatal period

*SBCE* social, behavioural and community engagement

**Table 7:** Top 10 newborn health SBCE research priorities

Rank	Newborn health research priority
1	Understand the determinants of the low adoption of family care practices and care-seeking behaviour for routine newborn care and for complications and improve the delivery of approaches to address these determinants
2	Find new approaches and improve the delivery of existing approaches to health education, to increase women’s knowledge and foster appropriate care seeking for complications during pregnancy, to improve maternal and newborn health outcomes
3	Find new approaches and improve the delivery of existing approaches to address gender inequity and to foster women’s empowerment and agency to improve their health and the health of their newborns
4	Find new approaches and improve the delivery of existing approaches (both in the community and at health-care facilities) to promote the provision of appropriate nutrition for newborns and children (including sick and vulnerable newborns) by caregivers and families to address hunger and malnutrition
5	Understand the determinants of early, exclusive and continued breastfeeding and find new approaches and improve the delivery of existing approaches to address these determinants, particularly at the community level
6	Understand the determinants of health-worker behaviours and performance (including both facility- and community-based health workers) that affect acceptability and utilisation of health services for newborns
7	Improve the delivery of approaches that engage community members in reducing the social and cultural factors that cause delays in accessing appropriate maternal and newborn care (including skilled care at birth)
8	Improve the delivery of community-based newborn care approaches that impact on family care practices for the newborn, particularly in areas with low rates of facility-based births and skilled care at birth
9	Find new approaches and improve the delivery of existing approaches to improve family care practices of preterm and low birth-weight babies
10	Find new approaches to foster effective male involvement in efforts to increase maternal, newborn and child well-being and access to care

*SBCE* social, behavioural and community engagement

**Table 8:** Top 10 child health SBCE research priorities

Rank	Child health research priority
1	Improve the delivery of SBCE approaches by community health workers for increased impact and scalability
2	Understand the determinants of the adoption of beneficial family care practices and care-seeking behaviour for infectious diseases among vulnerable populations
3	Understand the determinants of the health service delivery factors (e.g. health worker behaviours, service quality, and financial and opportunity costs) that influence community demand for and satisfaction with child health services and find new approaches and improve the delivery of existing approaches to increase utilisation of these health services
4	Improve the delivery of approaches to support family, community and health system actions to foster healthy early childhood development for all children, including children with disabilities
5	Find new approaches and improve the delivery of existing approaches to address gender inequity and to foster women’s empowerment and agency to improve their health and the health of their children
6	Improve the delivery of health education and counselling interventions to increase the uptake and maintenance of beneficial family care practices for maternal, newborn and child health
7	Understand the determinants of maintaining exclusive breastfeeding for the first 6 months and of maintaining breastfeeding for 2 years
8	Understand the determinants of optimal early childhood nutrition and growth, with attention to the role of gender and household dynamics, and find new approaches to address these determinants
9	Find new approaches for community participation and social accountability that improve access to and quality of care for newborns and children
10	Understand the determinants of maternal depression and assess its impact on women’s and children’s health and development

*SBCE* social, behavioural and community engagement

**Table 9:** Top 10 cross-cutting MNCH SBCE research priorities

Rank	Cross-cutting MNCH research priority
1	Improve the delivery of SBCE approaches to achieve universal health coverage of MNCH services for the most vulnerable populations
2	Understand the determinants of optimal uptake of maternal, newborn and child self-care and family care practices, and optimal care-seeking behaviour
3	Understand the determinants of health-worker behaviours and performance (including facility- and community-based health workers) that affect acceptability and utilisation of MNCH services and improve the delivery of approaches to address these determinants
4	Improve the delivery of approaches that address social norms (particularly gender norms) that cause delays in accessing appropriate MNCH care
5	Understand the determinants of optimal uptake of life-saving and other critical commodities for MNCH
6	Find new approaches and improve the delivery of existing approaches to scale up community mobilisation and engagement and to integrate them into healthcare services and other government systems for MNCH
7	Find new approaches and improve the delivery of existing approaches to enable effective learning, including peer-to-peer learning, engagement and empowerment of women, families and communities to foster behaviour change, health literacy and agency to improve MNCH
8	Improve the delivery of approaches to strengthen the integration of SBCE interventions into other services delivered by community health workers and community-based organisations to foster increased use of health services and improved MNCH
9	Find new approaches to measure emerging SBCE priorities (i.e. social inclusion, vulnerability, multi-sectorality and integration, and community capacity) to inform planning, programming and priority setting for MNCH
10	Understand the determinants and find new SBCE approaches to address women’s, newborns’ and children’s vulnerability to violence and injury

*MNCH* maternal, newborn and child health, *SBCE* social, behavioural and community engagement

## Discussion

In the research priorities that resulted from this exercise there are notable themes evident within each health area as well as themes that emerge across all areas.

Within the maternal health priorities, 8 of the top 10 priorities focused on research that improves the delivery of SBCE interventions and programmes that strengthen self-care, family care practices and care-seeking behaviour through SBCE interventions. This likely reflects the concern that the EWEC Survive objective (namely, ending preventable deaths and ensuring that women and newborns receive timely and appropriate care) still needs attention. The remaining two priorities were concerned with research on interventions that strengthen community capabilities and meaningful community involvement in shaping the responsiveness of health services to needs.

Eight of the top 10 priorities for newborn health focused on research on SBCE interventions that strengthen care practices, including breastfeeding, and care seeking. As with maternal health, this also appears to be very much linked to the EWEC Survive objective. Nonetheless, addressing gender inequity, women’s empowerment and effective male involvement were also prioritised, particularly in relation to how these can increase a woman’s ability to care for herself and her children.

Six of the top 10 child health priorities focused on improving the delivery of services and programmes but, compared to the other health areas, more emphasis was placed on understanding determinants of service utilisation, breastfeeding practices and nutrition practices. Health education and community participation were mentioned as interventions to improve care practices and care seeking. The child priorities also included health issues not addressed in the other health areas, namely early child development and maternal depression and its impact on child health and development.

In the cross-cutting area, there was a similar emphasis on how SBCE interventions improve the implementation of services and programmes, but there was more emphasis on the uptake of MNCH services and use of life-saving and other critical commodities. Also highlighted was a need for the better integration of SBCE approaches into programmes, including addressing all the levels of a socio-ecological model [13] (individual, family, community, services and policy). In a similar vein, experts said that an integrated view of health service delivery that addresses community-based and facility-based services is important, as is health systems thinking that links governance, commodities supply and human resources [14].

Across all health areas a recurrent theme was improving the delivery of existing programmes or known interventions. Out of the top 10 priorities, 8 each in maternal and newborn health, 6 in child health and 5 in cross-cutting focused on the delivery domain. We interpreted this as a clear call for improved implementation science for SBCE.

Throughout the different stages of the prioritisation process and across health areas, it was consistently highlighted that SBCE interventions are strongly linked to the context in which they are implemented. This reinforces what is found elsewhere in the literature, namely that SBCE interventions should be context specific and responsive to the local determinants, settings and populations (e.g. addressing the needs of rural settings) [15]. Because of this, there is a need for research on how SBCE interventions might vary across a range of different contexts. There was also consistent interest in promoting equity through SBCE research that helps overcome disparities in service access, quality and outcomes faced by specific populations that are marginalised, disadvantaged or vulnerable, such as adolescents, small and sick newborns, children with disabilities, and populations in humanitarian and emergency contexts.

Experts also emphasised the need to strengthen the methods for SBCE research. While evidence reviews have called for more robust research on programme effectiveness [5, 6] and the use of mixed-methods impact evaluations [4], the results present a call for more reflection on how to use causal chain analysis and process evaluation techniques to provide a more in-depth understanding of SBCE and how change occurs over time.

### Policy implications

This study has produced a research agenda that defines the top priorities globally for research on SBCE interventions to improve MNCH in the period up to 2030. It contains four sets of priorities for SBCE interventions to improve maternal health, newborn health and child health, and cross-cutting interventions that improve all three of these health areas, focusing especially on areas of women and community empowerment and participation in health programmes, self-care and family care practices, care seeking, and social determinants of health.

To our knowledge, this is the first specific priority setting exercise for research on SBCE interventions to improve MNCH and therefore provides clearer guidance to MNCH stakeholders on the priorities for SBCE research than has previously been available. The research agenda offers guidance to policy-makers and donors to direct their investments to the areas of SBCE research that are most needed to achieve the transformative aims of the EWEC Global Strategy to strengthen agency, build capacity and promote equitable health outcomes over the life course. Similarly, it provides guidance to researchers to undertake research that is aligned with the defined priorities.

Future work on MNCH topics should shed light on optimal research design, priority outcomes and measurements, core intervention delivery and implementation components, and the documentation of other factors, such as context, to further enhance understanding of how to conduct more impactful SBCE research. Past efforts to review the evidence base for SBCE interventions and their contribution to key MNCH outcomes revealed a lack of consistency in outcome measurements across studies and limited information available on important contextual and implementation considerations [5, 6].

Better reporting of implementation and contextual issues [16] and improved evidence synthesis and global guideline methods [17] will also contribute to a more coherent production line of primary research to evidence and, ultimately, increased inclusion of these interventions in policy recommendations and programmes.

### Strengths and limitations

The participation of experts from different regions of the globe and the mix of expertise that contributed to defining the priorities are an important strength of this exercise. A limitation of the CHNRI method is that is it ‘expert-focussed’ and the results of the exercise may represent a limited range of perspectives [18], typically biasing towards the perspective of researchers [9, 19]. To address this, the number of programme experts invited was increased relative to the number of researchers. The resulting balance in representation of researchers and programme experts is a strength of this exercise in that it captures the views of those who generate evidence and those who need and use evidence to inform practice and policy in programmes to improve MNCH. The strong representation of research priorities with an implementation focus may reflect the decision to invite a greater proportion of programme experts.

The use of online surveys with invitations sent to a large pool of experts was also intended to capture a broad range of perspectives but may have resulted in the exclusion of some experts who had limited access to technology. However, more than half of the experts who proposed and scored research priorities were based in low- and middle-income countries, indicating good representation of experts from contexts where improving MNCH is a priority. The language of the survey may also have limited the involvement of experts who work in languages other than English, Spanish or French.

Participants were requested to score up to 30 research priorities in their health area, rather than the whole set of priorities as is more typical in research priority setting exercises using the CHNRI method; this was done to increase response rates yet resulted in some variance in the number of times different research priorities were scored, as indicated in Table 5. This was a trade-off because a longer list of priorities may also have resulted in a lower response rate. Nevertheless, this approach still allowed us to obtain a good range of experts’ collective views for input into the consensus-building exercise to agree on the final set of research priorities.

Finally, it was challenging to prepare the set of research priorities for scoring and, in some instances, the distinction between two or more similar priorities was subtle. Hence, consultation with a small expert group was employed to finalise the top 10 research priorities using a qualitative and consensus-based approach. A mix of researchers and programme experts were engaged in this final consultation, including representatives from ministries of health and other intended end-users of the agenda that resulted from this exercise. Their perspectives were vital for ensuring a consolidated and distinct set of high-importance research priorities that were relevant for a range of end-users. The mixed-methods approach helped to strengthen the prioritisation process and the validity of its outcomes.

## Conclusions

If progress toward the Sustainable Development Goals, the EWEC Global Strategy and primary healthcare is to be accelerated, investment in SBCE interventions, which support building the capacities and rights of individuals, families and communities as powerful agents of their own health, must increase. However, an undefined research agenda that does not adequately respond to global or country priorities may impede the optimal uptake of SBCE interventions within country strategies. The research agenda on SBCE interventions to improve MNCH that has resulted from this exercise reflects the input of the range of researchers and programme experts. As such, it provides guidance on the research felt to be most needed to address important knowledge gaps on SBCE interventions for MNCH and it defines important considerations on how to advance this research to improve MNCH outcomes.

Stakeholders are strongly encouraged to consider these priorities to guide future investment in SBCE research. WHO is committed to working with global and country-level partners to take the identified priorities and insights and use them to develop more specific research questions and optimal research methods to answer them. It will be helpful to develop these plans in the context of specific country programmes and to engage with national level stakeholders to implement and use the results presented in this paper. These global research priorities and the approach described herein to develop these priorities can provide a blueprint for similar processes to be undertaken at regional and national levels.

Taking maximum advantage of the new research agenda will require continued collaboration to track the implementation of these research priorities and to synthesise the resulting evidence base.

Box 1 Categories of experts proposing SBCE research priorities for MNCH

**Table:** 

● Researchers	
● Programme experts:	
○ Ministries of health and selected other ministries of 81 high-burden countries	
○ Non-government organisations (international and national)	
○ For-profit private sector	
○ Technical assistance agencies	
○ Donors, funding agencies and foundations	
○ Professional organisations (international and national)	
○ Healthcare providers	
○ United Nations agencies (headquarters, regional and national)

Box 2 Scoring criteria

**Table:** 

** Health and social impact:** Will the research priority lead to interventions that have important health and social impacts for maternal, newborn and child health?	
** Equity:** Will the research priority provide benefits that improve health and social equity?	
** Feasibility/answerability:** Can the research priority plausibly be done and yield results within the timeframe for the research agenda	
** Overall importance:** Do you feel that the research priority is important?	
Participants were asked to indicate priority by assigning the following scores for each criterion:	
● 0 if the research priority does not meet the criterion	
● 1 if the research priority does not clearly meet or not meet the criterion	
● 2 if the research priority meets the criterion

## Data Availability

The datasets used and/or analysed during the current study are available from the corresponding author on reasonable request.

## Abbreviations

CHNRI: Child Health and Nutrition Research Initiative
EWEC: Every Woman Every Child
MNCH: maternal, newborn and child health
SBCE: social, behavioural and community engagement
